# Skin Diseases and Syphilis

**Published:** 1894-06

**Authors:** 


### SKIN DISEASES AND SYPHILIS.

EDITED BY DR. M. B. HUTCHINS.

### A CASE OF DESQUAMATIVE SCARLATINIFORM ERYTHEMA WITH A PROLONGED COURSE. (*Archiv für Dermatologie und Syphilis*, 1893, Heft 4.)

Under this title Török describes this case: A man fifty years old, who had always enjoyed good health and whose family history was good, had cut himself while shaving, some fourteen days before he came under observation. No application was made to the cut. The patient was not taking any drug. Two days after the injury he noticed a feeling of tension in the skin of the face, and on the next day noticed that it was red. In the course of a week the disease had spread all over the body. At the time of the first examination it was found that the essential features of the disease were redness and scaling. Over the whole scalp was a fine, meal-like scaling; the face, the neck and the ears were of a bright red color and covered with fine scales, which were especially well marked in the folds of the skin. The skin of the chest and of the back was bright red, with faint, darker colored spots scattered irregularly over it; a fine scaling was to be seen here also. The extensor side of the arm was red and the scales were larger—one half centimetre in diameter. Upon the flexor side of the arm were large irregularly-shaped red spots, disposed without regularity and covered with scales. Upon the thenar portion of the hand and under the wrist the skin was thickened and exfoliated in large lamellæ, leaving a red surface. In the interphalangeal folds, on the side of the fingers and at the bend of the phalangeal joints, the skin was red and smooth, while about the borders one could see a fringe of epidermis. Only lanugo hairs were left on the trunk and extremities, and it could be seen that the epidermic exfoliation had involved their follicles. On the belly and sacral region there was no diffuse redness, only red spots which scaled. The skin of the penis was of normal color with some scaling at the scrotal junction. The scrotum was pale red with fine scales. The lower extremities were affected in a similar manner to the upper, but to a lesser extent. About two months after the beginning of the disease the nails became rough and the hair began to fall out. The skin never showed any infiltration and the temperature was normal. The treatment consisted of baths and various ointments, of which those containing salicylic acid seem to have done most good. At the end of five months the patient had recovered.—*Brit. Jour. Derm., April*, 1894.

---

### COCAINE AS A LOCAL ANESTHETIC.

*Monatsh. fur Prak. Derm.* XVIII., No. 7, quoting Moraga, International Dermatological Congress, 1892, Vienna, gives his conclusions as follows:

1. Cocaine dissolved in distilled water loses a great part of its efficacy after some time.

2. Aqua laurocerasi should be used as a solvent instead of distilled water.

3. Cocaine is an excellent anesthetic (local).

4. The intensity and extent of anesthesia varies according to the dose, complete in many, incomplete in other cases.

5. Its action depends upon the susceptibility of the patient, small doses sometimes acting intensely, sometimes the reverse.

6. Generally a certain tolerance is established after some time, but always gradually and without the production of a decided cocainismus.

7. One should never begin with more than one-third to three-fifths of a grain, and never exceed one and one-half grains.

8. Generally those of lymphatic or nervous temperaments, women, children and those weakened from any cause are more susceptible.

9. Constitutional effects, as chilling of the extremities, difficulty of breathing, palpitation, etc., can only (?) be produced by doses exceeding one and one-half grains.

10. Cocaine produces no "bad after-effects." If one must stop its use it is because only of the immediate effect.

11. He believes that its employment is obligatory with every physician who understands its use.

12. Its use has no danger so long as the prescribed dose is not exceeded.

---

### RED BLOOD CORPUSCLES AND THEIR HEMOGLOBIN IN SYPHILIS.

Hjelmann (*Finsk. Lakaresallsk, Handl. Bd.* 32) concludes that: Anemia appears in recent syphilis and reaches its maximum at the period of eruption. The blood resumes its normal constitution as the general symptoms disappear. Mercury in the usual doses has no influence in the production of this anemia.—*M. für Prak. Derm.* XVIII., No. 7.

---

### RUBBER PLASTER.

P. Cousin recommends the use of caoutchouc coverings, applied according to the method of Dr. Tenneson, in eczema. He seems to regard it as a panacea in all forms of the disease.—(*Thèse de Dermatologie*, 1893.)—*Dr. Johnston Jour. Cut. and Gen. Ur. Dis., May*, 1894.

---

### SULPHOPHENATE OF SODIUM.

Spiegler has obtained good results in prurigo with this drug in 5 to 10 per cent. ointment, using equal parts of vaseline and lanolin, as a base. It is non-toxic, provokes no irritation.—*Dr. Johnston, Jour. Cut. and Gen. Ur. Dis., May*, 1894.

---

### BENZOIN.

This drug in the form of the compound tincture, either in combination in ointment or pure, has been found to be of considerable service in the treatment of seborrheic eczema. Alone it is also of great value in chronic eczema of the eyelids and lips of any origin.—*Dr. Johnston, Jour. Cut. and Gen. Ur. Dis., May*, 1894.

